# Retrospective Analysis of HER2+ Breast Cancer Outcomes at a County Hospital: Do Published Outcomes Hold up in the Real World?

**DOI:** 10.7759/cureus.7937

**Published:** 2020-05-02

**Authors:** Serene A Tareen, Joshua Rodriguez, David Bolos

**Affiliations:** 1 Internal Medicine, Olive View - University of California Los Angeles (UCLA) Medical Center, Los Angeles, USA; 2 Hematology & Oncology, Olive View - University of California Los Angeles (UCLA) Medical Center, Los Angeles, USA

**Keywords:** her2-positive, breast cancer, pathologic complete response, hispanic population, breast cancer outcomes, neoadjuvant chemotherapy, comparing outcomes at county hospital, unequal access to health care, mammography

## Abstract

Introduction: Landmark trials repeatedly demonstrate that pertuzumab and trastuzumab plus standard chemotherapy have the best outcomes in human epidermal growth factor receptor 2 (HER2) positive breast cancer in the neoadjuvant, adjuvant, and metastatic setting. However, many of these multicenter landmark trials lack diversity and studied largely Caucasian populations. Our goal is to address this under-representation of minorities, and compare pathologic complete response (pCR) rates in our predominantly Hispanic population with HER2 positive breast cancer receiving the same neoadjuvant chemotherapy (NACT) at Olive View-UCLA Medical Center (OVMC) to that of pCR rates observed in the TRYPHAENA trial.

Methods: For this retrospective cohort study, we compiled a list of 53 patients aged 18 and older, 52 female and 1 male, with HER2 positive breast cancer identified by fluorescence in situ hybridization treated at OVMC from December 2015 to May 2018. Our population was 57% Hispanic, 13% white, 13% Filipino, 11% Asian, 2% black, and 4% other. The complete list included patients receiving standard neoadjuvant, adjuvant, and metastatic chemotherapy regimens. We analyzed 23 female patients with HER2 positive breast cancer staged I to IIIC, receiving standard NACT (docetaxel, carboplatin, trastuzumab, and pertuzumab). Metastatic HER2 positive breast cancer patients were excluded. The primary outcome studied was pCR rates after receiving NACT. pCR was defined as the absence of invasive cancer cells from tissue samples removed after surgery. Secondary outcomes measured were side effects of chemotherapy. pCR rates and side effects were compared to TRYPHAENA. Data regarding insurance status, breast cancer detection modality, and time to seek medical attention were recorded.

Results: 50% of our patients who received NACT achieved pCR. Our pCR rates mirrored those observed in the TRYPHAENA trial (51.9%). The most common side effect observed in our population was diarrhea. A higher proportion (37.5%) of our patients had liver function test (LFT) elevation compared to the TRYPHAENA trial (3.9%). Baseline LFTs were normal prior to treatment in 96% of patients. In terms of modality of detection, 70% were self-palpated, 26% were detected through routine mammography, and 4% were found incidentally. Average time from mass discovery to seeking medical attention was 3.4 months. Only 26% had medical insurance at diagnosis. Although not included in our study, 28% of our patients were initially diagnosed with stage IV metastatic disease.

Conclusion: Our study found that pCR rates in our primarily Hispanic population compared well to the response rates observed in landmark trials with largely Caucasian populations. Genetic variations in chemo-sensitivity may have a minimal influence on cancer care outcomes in this population.

## Introduction

It is well known that global healthcare disparities among various socioeconomic backgrounds exist, and these differences have been broadly attributed to unequal access to appropriate healthcare. Particularly, variations in cancer outcomes have been largely explained by limitations in healthcare education, access to preventive screenings, vaccination, surgery, radiation, and advanced chemotherapy [1]. de Souza et al. compared cancer care in low, middle, and high-income countries and highlighted some startling statistics that illustrate the gravity of this issue. For example, in the low-income country of Kenya where only 4.7% of the gross domestic product (GDP) is distributed toward healthcare costs, only 18 of the 52 essential cancer medicines defined by the World Health Organization (WHO) are available [2]. The mortality-to-incidence ratio (MIR) of cancer is an indicator of the efficacy of a nation’s cancer control programs, and there is a positive association between low-income healthcare systems and higher MIRs [3]. Kenya’s MIR of cancer is 0.78 [1]. On the contrary, in high-income countries, such as the United States where healthcare spending accounts for 17% of the GDP, the MIR of cancer is 0.36 [1]. In the United States, cancer screening, hepatitis B and HPV vaccination, imaging, surgery, radiation, and access to advanced chemotherapy are widely available [1]. However in terms of breast cancer, according to the American Cancer society, differences in mammography screening rates in the United States persist primarily due to lack of insurance in vulnerable populations [4]. Data gathered from 2015 showed that only 31% of the uninsured and 46% of immigrants reported having a mammogram in the last two years, as compared to 68% of those with health insurance [4]. Therefore, despite the United States being in the top 20th percentile of the WHO's list of most efficacious healthcare systems by country, inequities continue to remain [5]. In summary, there are poorer cancer care outcomes in lower income countries with predominantly minority populations, and it is unclear if these differences are due to racial differences in the underlying disease and/or response to medical therapies or due to decreased healthcare literacy and lower funding towards healthcare. Therefore, we undertook a study to see if there was ethnic variation in response to herceptin-based chemotherapy in our hospital's largely Hispanic patient population.

Pertuzumab and trastuzumab are monoclonal antibodies that bind to different sites of the human epidermal growth factor receptor 2 (HER2), but together have a synergistic cytotoxic effect on HER2 over-expressed cancer cells [6]. Landmark trials have repeatedly demonstrated that pertuzumab and trastuzumab plus standard chemotherapy have the best outcomes in HER2-positive breast cancer [6-8]. The NeoSphere trial demonstrated that patients with HER2-positive breast cancer treated with pertuzumab and trastuzumab plus docetaxel in the neoadjuvant setting had higher pathologic complete response (pCR) rates than those treated with trastuzumab plus docetaxel [6]. The primary objectives of the TRYPHAENA trial were to assess the cardiac tolerability of neoadjuvant pertuzumab and trastuzumab given with anthracycline containing or anthracycline free standard chemotherapy in HER2-positive breast cancer, and compare pathologic response rates among the different treatment groups [9]. The CLEOPATRA trial was a phase III trial that looked at the combination of trastuzumab and pertuzumab and taxane in women with HER2-postive metastatic breast cancer [7]. However, despite the large population included in these multicenter studies, ethnic minority groups were underrepresented [7, 9]. Notably, the patients studied in the TRYPHAENA trial were 76.4% white, 18% Asian, 4% black, and 1.3% other [9]. This under-representation of minorities has prompted further investigation to evaluate if similar outcomes can be seen among black and Hispanic populations.

Villarreal-Garza et al. analyzed Hispanic women with HER2-positive breast cancer in Mexico treated with trastuzumab-based neoadjuvant chemotherapy (NACT), and showed that pCR rates mirror the rates of other ethnicities and further concluded that healthcare access to appropriate therapy rather than ethnicity has the greatest influence on breast cancer prognoses [10]. Another noteworthy study by Killelea et al. used the National Cancer Database from 2010 to 2011 to identify potential racial differences in women being treated for stage I to III breast cancer [11]. This study found that NACT was given more often to black, Hispanic, and Asian women than to white women [11]. This finding was thought to be secondary to diagnoses at more advanced stages, as well as a higher percentage of triple negative and HER2-positive breast cancers in the black, Hispanic, and Asian women [11]. Additionally, in women who had triple-negative and HER2-positive breast cancer, pCR rates were lower in black women as compared to other ethnic groups [11]. Whether these findings were attributed to genetic variations in chemo-sensitivity or differences in socioeconomic background could not be established.

Information regarding breast cancer outcomes particularly in the Hispanic population is limited. Our goal is to address this under-representation of minorities, and compare pCR rates seen in our predominantly Hispanic population at Olive View-UCLA Medical Center (OVMC) being treated for HER2-positive breast cancer with those who were treated in TRYPHAENA trial. Our secondary aim is to compare adverse effects seen in our population with those seen in the TRYPHAENA trial. We also report data regarding insurance status, breast cancer detection modalities, and time to seek medical attention to better understand barriers to treatment.

## Materials and methods

For this retrospective cohort study, we analyzed 53 breast cancer patients who were aged 18 and older, and classified as having HER2-positive breast cancer identified by fluorescence in situ hybridization (FISH) or immunohistochemistry (IHC) testing. The patients were treated at OVMC from December 2015 to May of 2018. The patient demographic panel was predominantly Hispanic (57%), with the remaining comprising 13% white, 13% Filipino, 11% Asian, 2% black, and 4% other. Of the total cohort of patients, 23 (43%) were treated for stage I to IIIC breast cancer and had received standard NACT with docetaxel, carboplatin, trastuzumab, and pertuzumab (TCHP) as had been prescribed in the TRYPHAENA trial. The primary outcome studied was pCR rates defined as the absence of invasive cancer cells from tissue samples removed after surgery [12]. Secondary outcomes reviewed were adverse effects (AEs) including diarrhea, neuropathy, neutropenic fever, mucositis, liver function abnormalities, and electrolyte derangements. Finally, we record data regarding insurance status at the time of diagnosis, the mode of discovery of initial breast mass, and the time it took from discovery of palpable breast mass to seeking medical care in our cohort of patients.

## Results

Among our 23 patients receiving NACT, the pCR rates mirrored the rates observed in the TRYPHAENA trial [9]. Some 50% of patients who received the standard NACT had pCR as compared to 51.9% observed in the TRYPHAENA trial. Notably, in our cohort receiving NACT, Hispanic patients had pCR rates at 64%. The slightly higher pCR rates were attributed to the lower number (n=24) of patients studied as compared to the 225 studied in the TRYPHAENA trial [9]. Similarly, as observed in the TRYPHAENA trial, the most common side effect observed in our population was diarrhea (Table 1) [9]. Of note, a higher percentage of our patient population experienced liver function test (LFT) elevation, 37.5% as compared to 3.9% in TRYPHAENA. Baseline LFTs were normal prior to treatment in 96% of patients.

**Table 1:** Adverse effects of NACT in the OVMC population. NACT, neoadjuvant chemotherapy; OVMC, Olive View-UCLA Medical Center; LFT, liver function test

Side effects	Number (%)
Diarrhea	21 (87.5%)
Electrolyte derangements	14 (58.3%)
Neuropathy	9 (37.5%)
LFT abnormalities	9 (37.5%)
Mucositis	5 (20.8%)
Neutropenic fever	5 (20.8%)

**Figure 1:**
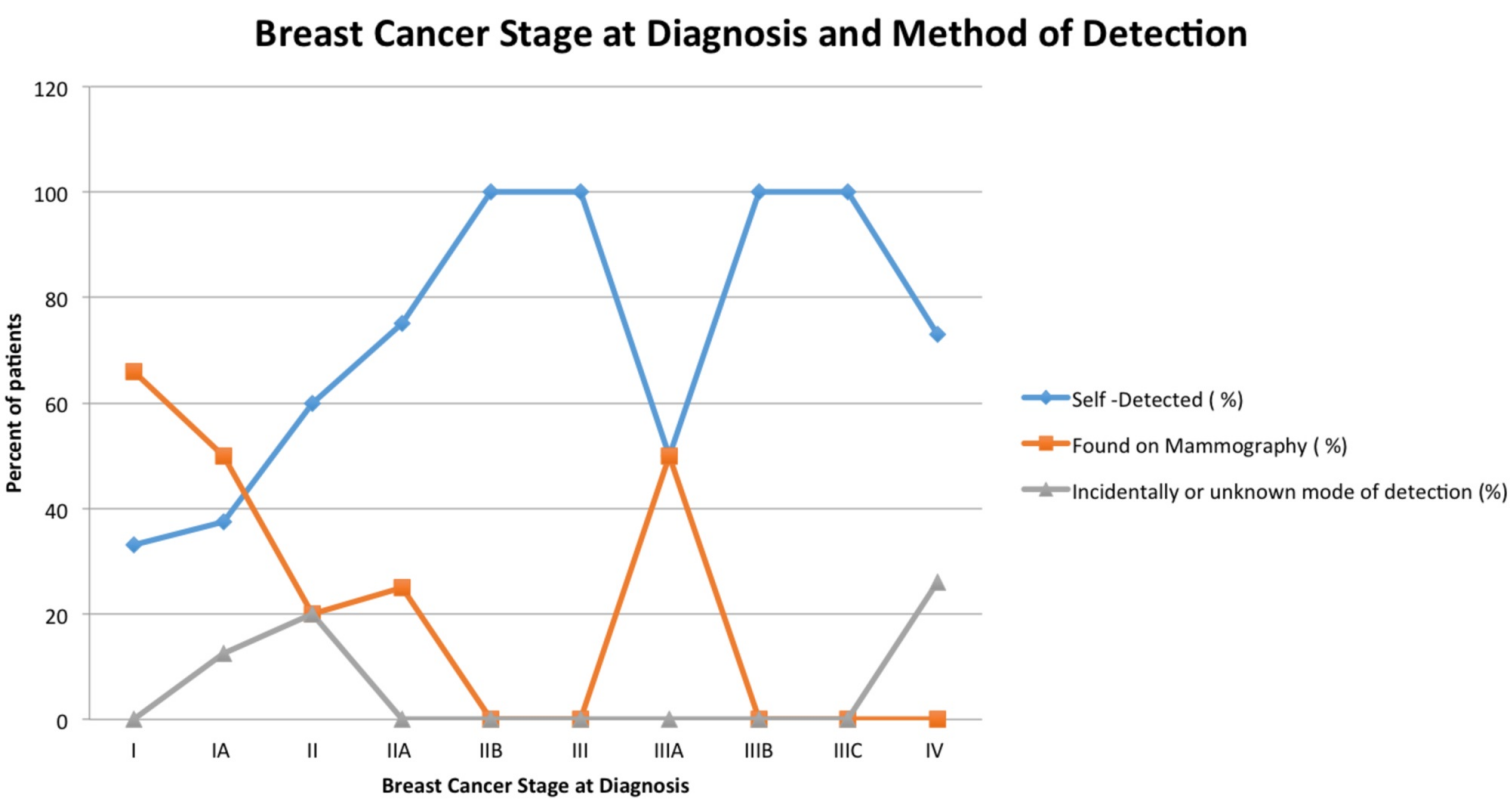
Breast cancer stage at diagnosis and method of detection. The complete list of 53 patients was stratified based on stage at diagnosis (x axis), and mode of detection reported as a percent by group (y axis).

With regard to mode of discovery of initial breast mass, 70% of patients had discovered a breast lump on self-examination, 26% were detected through routine mammography, and 4% were incidentally found on other imaging studies. Figure 1 illustrates that self-detected breast cancers more frequently represent advanced stages of breast cancer. It also demonstrates that mammography more frequently detects earlier stages of breast cancer. 

**Figure 2:**
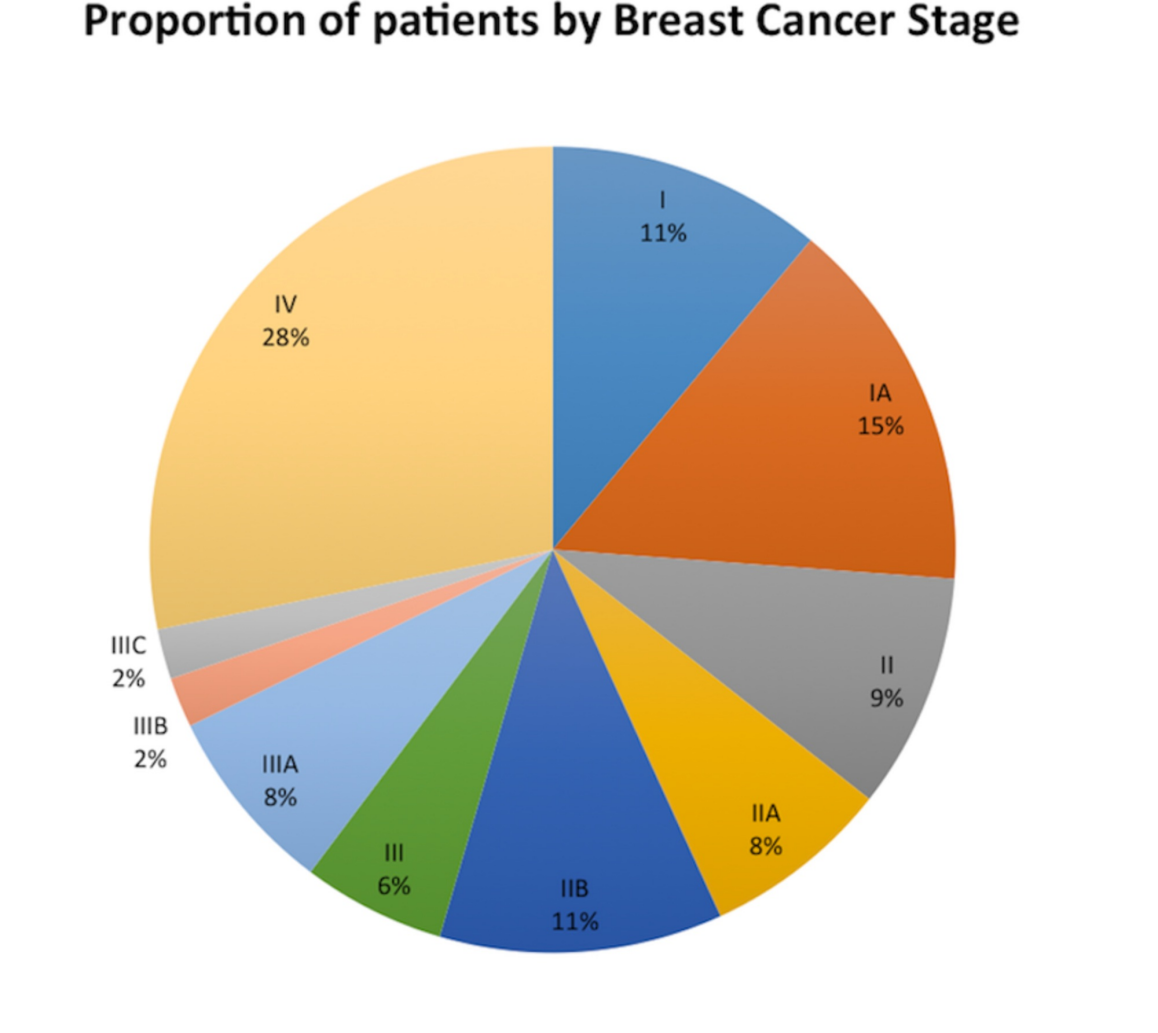
Breast cancer stage at diagnosis in the OVMC population. The above pie chart illustrates the proportion of patients in each breast cancer stage at diagnosis for our complete list of 53 patients. OVMC, Olive View-UCLA Medical Center

Among those whose breast masses were discovered by self-examination, 34% of women were below the recommended age of 50 as defined by the United States Preventive Task Force (USPTF) for screening mammography [13]. The average time from discovery of mass to seeking medical attention was about 3.4 months. Of the entire cohort, 26% of patients had medical insurance at the time of diagnosis. Notably, 28% of our patients were initially diagnosed with stage IV metastatic disease (Figure 2). 

## Discussion

By demonstrating that our patients had roughly the same pCR rates as those observed in landmark trials, our study suggests that genetic variations in chemo-sensitivity may have a minimal influence on cancer care outcomes in this population. One of the most noteworthy findings of our study was that 70% of our patients’ breast cancer was self-detected, and only 26% were identified through mammography. In contrast, national averages show a more even distribution. A study published in the Journal of Women’s Health used a U.S. national representative self-report health survey to ask 361 female breast cancer survivors diagnosed with breast cancer between 1980 and 2003 how their tumors were identified. The results of this study were that 43% were found by mammography, 43% were found through self-breast exam, 13% by clinical breast exam, and 1% by other [14]. This difference in the mode of detection among our patient population could potentially be explained by the fact that only 26% of our patients had insurance at the time of diagnosis. In addition, when our total cohort of patients were stratified based on the stage at diagnosis, we found that earlier stages of breast cancer were more frequently found on mammography, whereas self-detected breast cancers were more likely to represent advanced stages of breast cancer (Figure 2). Shen et al. specifically analyzed three large breast cancer screening trials to evaluate the role of detection method in predicting breast cancer survival [15]. This study accounted for lead time bias by comparing tumors diagnosed at the same stage, and found a survival benefit for patients with screen-detected breast cancers compared with those with breast cancers detected symptomatically [15]. Notably, when they adjusted by tumor stage, patients with symptomatically detected cancers had a 53% (95% CI = 17% to 100%) greater hazard of breast cancer-related death than patients with screen-detected breast cancers [15]. The aforementioned finding is of particular significance when applied to our study because it suggests that method of detection is an independent prognostic factor for breast cancer survival [15]. Given that our study was a short retrospective analysis, we were not able to compare mortality rates among stages. However, it is important to note that 28% of our total cohort of patients was initially diagnosed as stage IV disease. This is in contrast to the 6%-10% of all breast cancers being initially diagnosed as stage IV in the general population according to the American Cancer Society [16]. The inherent aggressiveness of HER2-receptor positive breast cancers may partially account for the higher percentage of stage IV disease at diagnosis observed in our patient population. However, the fact that at least 70% of the breast cancers were self-detected, unequal access to healthcare in our patient population cannot be understated.

According to the 2018-2020 Cancer Facts and Figures for Hispanics/Latinos by the American Cancer Society, Hispanics have the lowest likelihood among all ethnic groups to have health insurance [17]. Specifically, 25% of Hispanic adults aged 18-64 in 2016-2017 were uninsured, as compared to 9% of non-Hispanic whites [17]. Since the passage of the 2010 Affordable Care Act (ACA) and expansion of Medicaid, there has been a 36% decrease in uninsured working Hispanics from September 2013 to June 2014. For states that expanded Medicaid, there was a 50% decrease in uninsured Hispanics [17]. However challenges persist, as some states with large Hispanic populations, namely Texas and Florida, did not expand Medicaid coverage [17]. In addition, part of this discrepancy was attributed to Hispanics working in lower paying jobs, which are less likely to have employer-based healthcare coverage [17]. Language barriers were also addressed as a potential limiting factor towards enrolment. They found that after the first ACA enrolment period, 30% of Spanish-speaking Hispanics remained uninsured as compared to 19% of English- speaking Hispanics [17]. It was also noted, that enrolment to the ACA is not open to undocumented immigrants [17]. Given that many of our patients are undocumented, primarily Spanish speaking, and lower wage earners, these factors may explain the disproportionately high uninsured rate in our patient population.

Our side effect profile with the exception of LFT elevation was similar to that seen in the TRYPHAENA trial [9]. It was hypothesized that perhaps this difference could be attributed to higher incidence of fatty liver disease due to diet in our patient population. Unfortunately, the frequency of diabetes and hyperlipidemia was not recorded and may have supported the notion that metabolic syndrome contributed to greater liver function abnormalities. However, it is worth noting that 96% of our patients’ baseline LFTs were normal. A study by Phan et al., discussed variations in genes encoding drug metabolizing enzymes among different ethnic groups which result in different pharmacokinetics and pharmacodynamics of anticancer drugs [18]. These genetic variations in metabolism could potentially explain the higher incidence of liver function elevations in our primarily Hispanic population. 

## Conclusions

Our study found that pCR rates in our primarily Hispanic population compared well to the response rates observed in a landmark trial with a largely Caucasian population. Genetic variations in chemo-sensitivity may have a minimal influence on cancer care outcomes in this population.
